# Xanthogranulomatous Appendicitis in a Child: Report of a Case and Review of the Literature

**DOI:** 10.1155/2013/498191

**Published:** 2013-09-04

**Authors:** Sura M. Al-Rawabdeh, Vinay Prasad, Denis R. King, Samir B. Kahwash

**Affiliations:** ^1^Department of Pathology and Laboratory Medicine, Nationwide Children's Hospital, Columbus, OH 43205, USA; ^2^Department of Pediatric Surgery, Nationwide Children's Hospital, Columbus, OH 43205, USA

## Abstract

Xanthogranulomatous inflammation is a well-described inflammatory process, which may involve any organ but is most frequently encountered in the gall bladder and the kidney. There are rare reports of xanthogranulomatous appendicitis (XA) in the adult population, but only one brief mention of such a diagnosis in a child. In this report, we describe the case of an 11-year-old boy who presented with clinical signs and symptoms of acute appendicitis necessitating appendectomy. Upon microscopic examination, the appendix showed the typical features of XA. To the best of our knowledge, this is the first well-described case XA in a noninterval appendix in a child. We also reviewed the limited medical literature on the subject.

## 1. Case Report

The patient is an 11-year-old boy who presented with a 1-day history of abdominal pain and emesis but no fever. His past medical history was unremarkable. In particular, there was no history of hemorrhagic problems or systemic disease. The family history was also unremarkable. On physical examination, he had persistent and well-localized right lower quadrant and right flank tenderness at the expected location of his appendix (McBurney's point). The laboratory findings were unremarkable. WBC was 4.9 K/microliter with the following differentials: 49% segmented neutrophils, 41% lymphocytes, 9% monocytes, and 1% eosinophils. There was no evidence of anemia or thrombocytopenia (hemoglobin = 13 g/dL, hematocrit = 36%, and platelet count = 291 K/microliter). A computed tomography scan (CT-Scan) of the abdomen showed an enlarged appendix without inflammation; however, ultrasound images showed a fluid-filled appendix with a diameter within the upper rages of normal. A subsequent physical examination revealed an increase in abdominal pain and tenderness, and, consequently, the patient underwent a laparoscopic appendectomy. 

**Figure 1:**
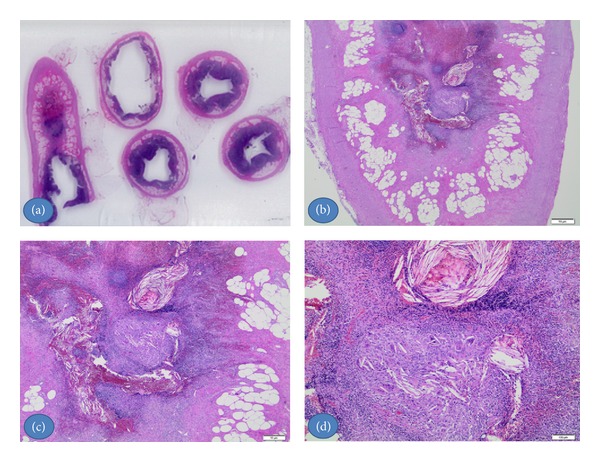
H&E stained section of the appendix. (a) and (b) Lower magnification shows the xanthogranulomatous focus in the tip of the appendix. (c) and (d) At higher magnification the typical appearance of xanthogranulomatous inflammation with it is component, the xanthoma cells, giant cells, cholesterol clefts, chronic inflammatory cells and hemorrhage.

**Figure 2:**
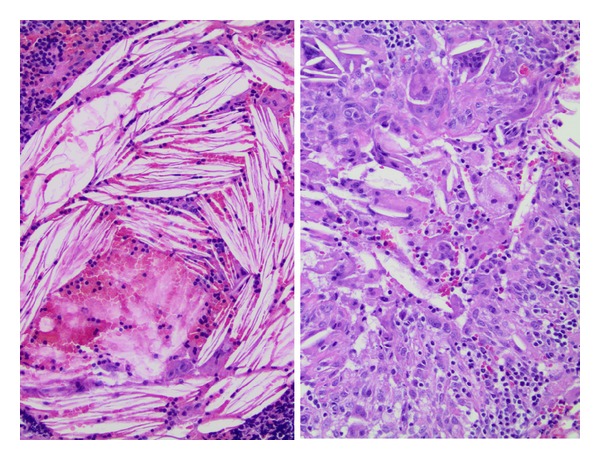
H&E stained section of the appendix. H&E stained section of the appendix on high power view of the numerous xanthoma cells, giant cells, and cholesterol clefts.

Gross evaluation showed a pink-tan appendix, measuring 8.3 cm in length and 1 cm in diameter. The serosal surface was unremarkable and cut surface demonstrated no fecalith. Microscopically, hematoxylin-eosin-stained sections of the tip of the appendix revealed numerous lipid-laden xanthoma cells in the mucosa (Figure 1) which were surrounded by lymphocytes and plasma cells admixed with multiple multinucleated giant cells containing cholesterol clefts (Figure 2). The rest of the mucosa showed patchy mild neutrophilic infiltration.

The postoperative clinical course was unremarkable. The patient was discharged home the following day and had an unremarkable physical examination on a follow-up visit three weeks later.

## 2. Discussion

Xanthogranulomatous inflammation is a rare form of chronic inflammation, manifested by the presence of lipid-laden macrophages admixed with lymphocytes, plasma cells, neutrophils, and often multinucleated giant cells with or without cholesterol clefts [1]. It was initially described in the kidney by Osterlind in 1944 [2]. It has also been reported in other organs, such as gall bladder, prostate, epididymis, ovary, urinary bladder, kidney, appendix, and others [1, 3, 4]. The exact etiology of xanthogranulomatous inflammation is uncertain. Proposed etiologies include defective lipid transport, immunologic disturbances, infection by low-virulence organisms, and lymphatic obstruction [5].

Cozzutto and Carbone noted that hemorrhage plays a major role in the development of foamy macrophages, postulating that the ingested erythrocytes and platelets at the bleeding site overwhelm the lysosomal system of the macrophages causing deposition of phospholipids which results in a foamy appearance of the macrophages [1].

Involvement of the appendix by xanthogranulomatous inflammation, or xanthogranulomatous appendicitis (XA), is a rare phenomenon with only 10 cases reported in the literature. Nine of these case reports were described in adults (mean age = 42.3 year, range is from 12 years to 78 years old). One study described the case of a 12-year-old boy who had XA in an interval appendectomy, and specimen was removed 6 weeks after an episode of acute appendicitis [6].

Guo and Greenson reviewed the histopathology result of all interval appendectomy specimens within a 4-year period and compared them with a control group of 44 patients who had acute appendicitis and appendectomy within 72 hours of symptoms onset [7]. Xanthogranulomatous inflammation was seen in 8 of 22 (36.4%) of the interval appendectomy cases, but none of the acute appendicitis group [7]. They concluded that delayed or interval appendectomy specimens often have a characteristic inflammatory pattern that includes granulomas, xanthogranulomatous inflammation, mural fibrosis/thickening, and transmural chronic inflammation [7]. Without the appropriate clinical history, these changes may be misinterpreted as Crohn's disease.

In 1993, Birch et al. published the first reported cases of XA [8]. McVey et al. added another case of XA to the literature just a year later [9]. Both authors suggested an association of the xanthogranulomatous response with long-standing appendiceal inflammation and the formation of appendiceal mass. 

Munichor et al. reported XA in a 37-year-old female who presented with typical signs and symptoms of acute appendicitis [5]. In that report, an electron microscopic examination of the appendix specimen showed electron-lucent lipid droplets of variable size in the xanthoma cells and in other cells [5]. The authors suggested that different types of cells could be damaged by a common mechanism which includes obstruction, hemorrhage inflammation, and the resulting hypoxia [5].

In 2005, Chuang et al. reported a case of a 39-year-old man who presented with fever, right lower abdominal pain, and a mass [10]. A hemicolectomy was performed for suspected cancer and the pathological examination showed XA [10]. This case illustrated that xanthogranulomatous appendicitis may mimic a locally invasive cancer [10].

Omar et al. reported a rare case of XA, and cecal angiolipoma in a patient who presented with acute appendicitis and appendicular mass [11].

In 2011, Martínez-Garza et al. reported a case of XA and they also performed a literature review [3]. In their conclusion, they indicated that this diagnosis might be associated with inflammatory bowel disease [3].

The oldest patient diagnosed with XA was a 78-year-old Japanese man who presented with 2-month history of right lower quadrant abdominal pain [12].

Finally, Singh et al. reported a case of XA in a 21-year-old woman, who presented with abdominal pain and underwent surgery for suspected appendicitis [13]. They highlight in their conclusion the rarity of xanthogranulomatous inflammation in the appendix presenting as an acute event [13].

The classic microscopic pathologic appearance of XA demonstrates numerous lipid-laden macrophages (foam cells), abundant hemosiderin, and multinucleated giant cells, admixed with cholesterol clefts and mixed inflammatory cell infiltrate [14].

Several factors precipitating XA have been proposed as follows: organ obstruction, suppurative infection, hemorrhage, defective lipid transport, and local hypoxia which can trigger tissue damage within the involved organs, usually eliciting a microscopic response of the disease process [4, 5]. There appears to be a higher incidence of XA in interval appendectomies as noted by Guo and Greenson and seen in the cases by Singh et al., Chuang et al., and Mado et al. [6, 7, 10, 12].

Our case was remarkable for the finding of XA in a child with an acute (noninterval) presentation and also a CBC that was not suggestive of an acute suppurative inflammation.

## 3. Conclusion 

Xanthogranulomatous appendicitis (XA) is a rare clinical entity, particularly in the pediatric population. It may be encountered in specimens resected in the acute phase, but is much more common in interval appendectomy specimens. 

As observed in the present case and in previous reports, most patients with XA present with a picture of acute or subacute abdominal pain, and occasionally with a mass lesion.
